# Fabrication, Property and Application of Calcium Alginate Fiber: A Review

**DOI:** 10.3390/polym14153227

**Published:** 2022-08-08

**Authors:** Xiaolin Zhang, Xinran Wang, Wei Fan, Yi Liu, Qi Wang, Lin Weng

**Affiliations:** 1 School of Textile-Science and Engineering, Xi’an Polytechnic University, Xi’an 710048, China; 2 Key Laboratory of Functional Textile Material and Product, Xi’an Polytechnic University, Ministry of Education, Xi’an 710048, China; 3 Department of Chemical Engineering, Xi’an Jiaotong University, Xi’an 710049, China

**Keywords:** alginate fiber, preparation method, application properties

## Abstract

As a natural linear polysaccharide, alginate can be gelled into calcium alginate fiber and exploited for functional material applications. Owing to its high hygroscopicity, biocompatibility, nontoxicity and non-flammability, calcium alginate fiber has found a variety of potential applications. This article gives a comprehensive overview of research on calcium alginate fiber, starting from the fabrication technique of wet spinning and microfluidic spinning, followed by a detailed description of the moisture absorption ability, biocompatibility and intrinsic fire-resistant performance of calcium alginate fiber, and briefly introduces its corresponding applications in biomaterials, fire-retardant and other advanced materials that have been extensively studied over the past decade. This review assists in better design and preparation of the alginate bio-based fiber and puts forward new perspectives for further study on alginate fiber, which can benefit the future development of the booming eco-friendly marine biomass polysaccharide fiber.

## 1. Introduction

**Figure 1:**
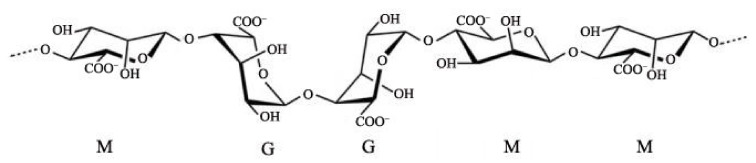
The structural schematic diagram of chain conformation and M/G block distribution of alginate molecular chain.

**Figure 2:**
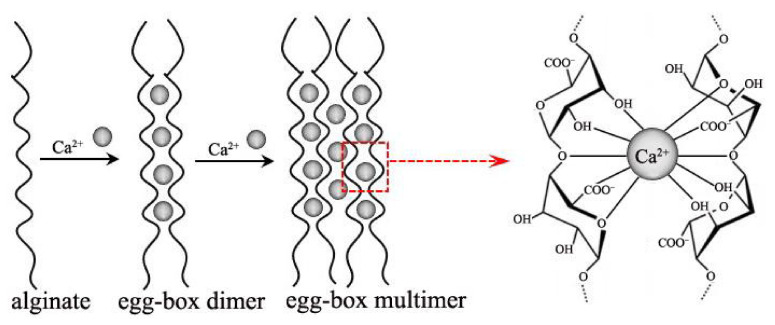
Egg-box junctions of calcium ion and the G block of alginate.

In recent years, alginate has become one of the most preferred materials as an abundant natural biopolymer [1]. Alginate is a linear polymer polysaccharide composed of β-D-mannuronic acid (M block) and α-L-guluronic acid (G block) jointed by 1,4-linkages, which is extracted from either brown algae or some genera of bacteria. The molecular chain is arranged in an irregular blockwise pattern of varying proportions of G-G, M-G and M-M blocks [2,3], shown in Figure 1. The percentage of M and G blocks and their distribution has an impact on the physicochemical properties of alginate, such as alginate of rich M units displays a flexible structure and better biocompatibility, while alginate of enriched G units exhibits a rigid molecular structure [4]. Alginate is known to be rich in carboxyl and hydroxyl groups distributed along the backbone, making it open to chemical functionalization and cross-linking treatment [5]. Typically, alginate can be cross-linked to form a hydrogel in the presence of divalent or trivalent metal cations, such as Fe^3+^, Al^3+^, Cr^3+^, Cu^2+^, Ba^2+^, Sr^2+^, Ca^2+^, et al. [6]. The gelation mechanism is the coordination between the carboxyl groups of alginate and the metal ions. Taking Ca^2+^ as an example, each calcium ion forms coordination bonds with two G units of the alginate molecular chain, which is called the egg-box structure [7,8], as shown in Figure 2.

**Figure 3:**
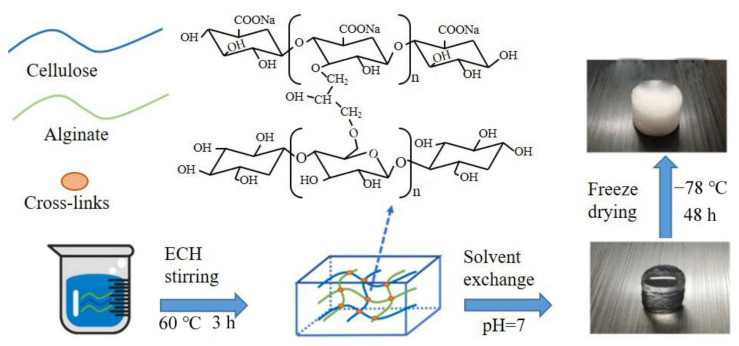
Fabrication of the alginate hydrogel and its porous scaffold by combining ionic cross-linking and freeze-drying techniques.

Based on the ability to gel with metal ions, different alginate-based materials with various morphology were developed, such as the porous scaffold [9,10,11], hydrogel [12,13], fiber [14,15,16], nonwoven fabric [17,18], membrane [19,20,21], and so forth. The common way to fabricate the alginate-based materials includes ion cross-linking [22], microfluidic spinning technique [23], freeze-drying [24], wet spinning technology [25] and immersive rotary/centrifugal jet spinning technique [26,27,28]. Recently, alginate hydrogel has been prepared by the freeze-drying technique or ionic cross-linking method. Commonly, the calcium salt solution was dropped into the homogeneous alginate solution dropwise to induce the cross-linking and form calcium alginate hydrogel. Moreover, the hydrogel can be also endowed with freeze-drying treatment to eliminate water, and then the porous scaffold is obtained. As displayed in Figure 3, Che et al. [11] used the alginate to cross-link with cellulose to fabricate the alginate-based composite hydrogel, which was then lyophilized to be the hydrogel porous scaffold. The pore diameter of the scaffold was precisely tuned by adjusting the lyophilization process parameters such as the lyophilization temperature and time. As well known, pores of the lyophilized scaffold can provide the oxygen and nutrient substance for the host cell to facilitate the growth of new tissue. Moreover, the alginate porous scaffold can be acquired without ionic cross-linking, just by the freeze-drying technique. In order to enrich the application properties of the alginate scaffold, some other polymers such as gelatin [29,30], chitosan [31,32] and collagen [33,34] were also incorporated into the alginate polymer to lyophilize and form the hybrid multi-functional lyophilized scaffold.

**Figure 4:**
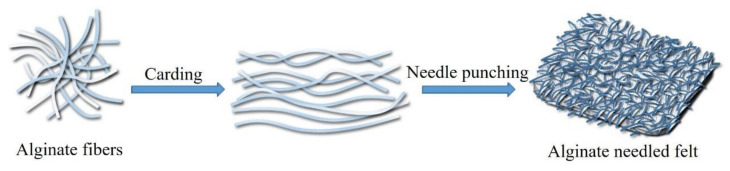
Preparation of the alginate nonwoven fabric by the acupuncture technique.

The alginate fiber is a research hotspot in the field of textile material, but the shortage of related reviews results in a lack of systematic reviews. Hence, this review focused on the fabrication and application properties of alginate fiber, which relied on the coordination between the metal ions exchanging the alkaline metal ions, usually sodium, and forming a strong bond with the G unit to precipitate fiber. The most classic way to obtain alginate fiber is either the microfluidic spinning or wet spinning technique [35,36,37,38,39], or electrospinning for fiber with less diameter requirement. The specific preparation process is described in the following text. The alginate nonwoven fabric can be prepared by the needle punching technology as shown in Figure 4, where alginate fiber is firstly combed into the fiber web and then the needled felt is obtained by the needle-punching. The alginate nonwoven fabric has multiple applications, such as wound dressing, tissue scaffolds, or facial masks due to its excellent hygroscopic property and water retention capacity [40].

There are several reviews on the alginate materials, which present the biomedical application properties of alginate hydrogel or the chemical modification of alginate polymer, but the review on alginate fibers is rare. However, the fiber has a broad application in the field of textiles and biomedical materials. It is urgent to give comprehensive knowledge of alginate fiber including the preparation method, the application characteristics and future progress, assisting researchers to better understand and develop novel alginate-based materials. Hence, the fabrication, physicochemical performance and different application properties of the alginate fiber are going to be introduced in detail in this review. The physicochemical properties included its mechanical performance, moisture absorption performance and biocompatibility, that correspond to the application.

## 2. Preparation Method of Calcium Alginate Fiber

### 2.1. Wet Spinning

**Figure 5:**
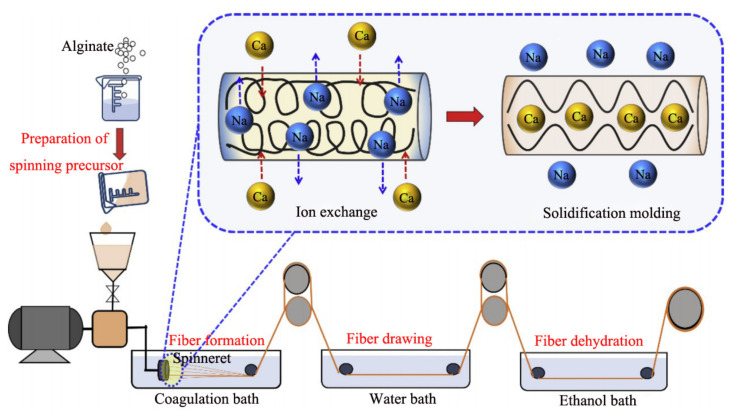
Schematic diagram of wet spinning process of calcium alginate fiber.

The wet spinning technique is a pioneering approach for preparing the alginate fiber and its schematic diagram is exhibited in Figure 5. The homogeneous alginate spinning solution extruded from the spinneret is introduced into a coagulation bath consisting of calcium salt solution to induce ion cross-linking and then form the primary fiber. The multiple draft rollers are installed in the coagulation bath to endow the primary fiber with a proper drawing ratio, which is beneficial for the improvement of fibrous mechanical performance. Moreover, to further enrich the application properties of alginate fiber, some researchers combined Ca^2+^ with other metal ions such as Zn^2+^, Ba^2+^, Cu^2+^, Al^3+^ and so forth to form the multi-metal ions coagulation bath [41]. There are some differences in terms of the chelation interaction of various metal ions with alginate molecules, resulting in various formation rates of fiber.

**Figure 6:**
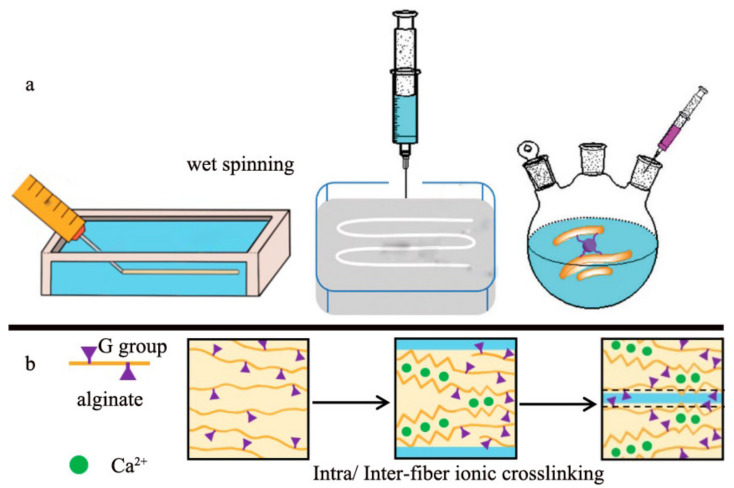
A mini modified version wet spinning device, (**a**) schematic diagram of the fiber spinning and (**b**) the formation mechanism of fiber.

The wet spinning device is large and is not versatile enough for the fabrication of alginate fiber with a special shape and function. Therefore, a modified version, mini wet spinning device was proposed and developed by some researchers [33,34] as displayed in Figure 6. During the fabrication, the alginate spinning dope in the syringe is extruded into the CaCl_2_ solution to finish the ion exchange and then the primary fibers will be obtained without further stretching. In addition, to enhance the mechanical performance and extend the application properties of alginate fiber, some other functional polymers or cross-linkers were added to the spinning dose to produce the hybrid fiber [40,42,43]. For instance, the catechol [44], quaternary ammonium chitosan (QAC) polysaccharide polyelectrolyte complex (PEC) [45] and the hydrolysis compound of vinyl triethoxy silane (VTES) [46] was incorporated in the alginate solution, respectively, to fabricate the composite fiber by the homemade wet spinning device. The introduction of those polymers can form the intermolecular cross-linking and thus improve the fibrous mechanical properties as well as endowing special functions such as the photo-thermal and antibacterial performance. Compared with the traditional wet spinning device, its modified version can realize the variety in fibrous function and structure. However, the lack of stretching dramatically decreased the mechanical properties of the alginate fiber.

### 2.2. Microfluidic Spinning

**Figure 7:**
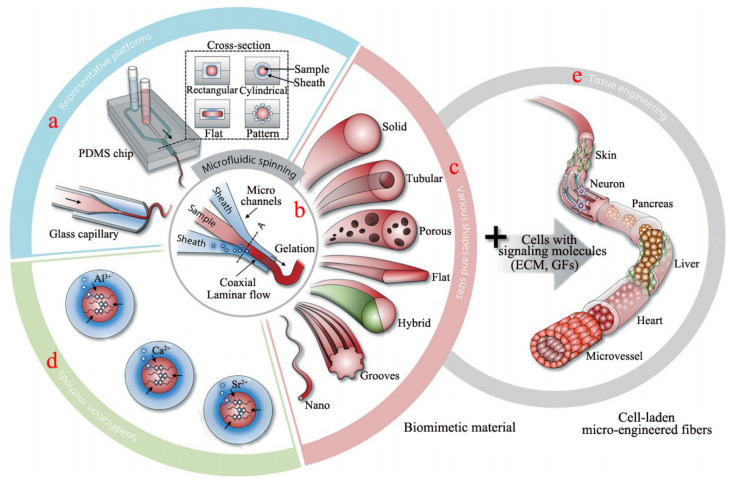
(**a**) The micro-channel device with various cross sections, (**b**) Overview of the microfluidic platforms composed of coaxial core and sheath fluids, (**c**) Anisotropic structure of alginate fiber fabricated by the MST, (**d**) Various metal cations cross-linked with alginate polymer to fabricate fiber and (**e**) The cell load micro-engineered fibers as the desired biomimetic material.

**Figure 8:**
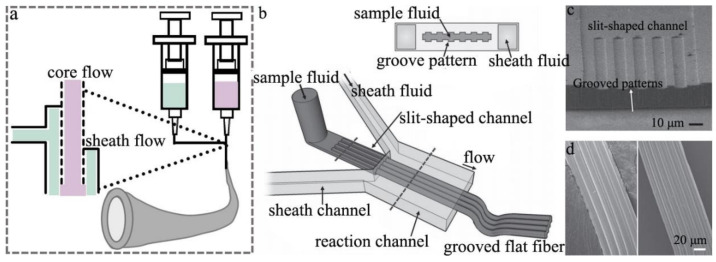
Schematic diagram of (**a**) microfluidic spinning system and (**b**) generating flat fibers with micro-grooves, SEM images of (**c**) the slit-shaped channel and (**d**) alginate fiber with grooved structure.

Based on the development of wet-spun alginate fiber, some researchers developed the microfluidic spinning device, which is an efficient and facile strategy to fabricate alginate fiber. The preparation of alginate fiber by the microfluidic spinning technique (MST) has received much attention recently, due to its eco-friendly, simple and effective implementation. That also makes MST a popular method to function as a microbioreactor, including the bio-chip. The schematic diagram of fabricating alginate fiber by MST is illustrated in Figure 7a,b, in which the core and sheath flows are the alginate and CaCl_2_ aqueous solution, respectively. The micro-channel can be endowed with various cross sections (Figure 7a), resulting in the generated fibers with various structures containing tubular, porous, flat, hybrid and hollow (Figure 7c) [47,48]. Calcium cation is the most common cation used to cross-link alginate to form fiber, but other metal cations including Ba^2+^, Al^3+^, Cu^2+^ and Zn^2+^, et al. (Figure 7d) can also coordinate with G units of alginate to form a fiber. Significantly, some cells with signaling molecules such as extracellular matrix or growth factors can be added to the sample solution to fabricate the multi-functional alginate fiber application in the tissue engineering scaffold including skin, liver, heart and microvessel (Figure 7e) [49,50]. The micro-channel of MST is mainly composed of the core and sheath micro-channel (Figure 8a,b), which can be made of the pulled glass tube and the stainless steel tube. Furthermore, the channel can also be produced by using the micro-electromechanical systems technology to engrave on the surface of the polydimethylsiloxane (PDMS) platform, which then creates the PDMS microfluidic spinning device with various topological constructions (Figure 8c). The method can endow the channel with a variety of parallel-grooved substrata with various pitches and depths, which is beneficial for producing the orientated-microgroove structure of alginate fiber (Figure 8d) [51]. There was a report on the tuning core-sheath flow rate to achieve the helical alginate microfiber as well as the incorporation of Fe_3_O_4_ magnetic substance via the MST [36].

The microfluidic spinning device not only can be used to fabricate the alginate microfiber with various structures but also offer the fiber with different functions by the addition of a bioactive small molecule substance, making it an ideal candidate for controlled release as the biomimetic material. Some other works have incorporated the bioactive substance such as the protein or cell in alginate solution to fabricate the multi-functional alginate fiber with various micro-structures including the flat, hollow and grooved anisotropic structure via the MST, that was used as the micro-vascular application in the field of tissue engineering [47,52,53]. Especially, the micro-grooved flat fiber has not only been used to guide the morphogenesis of various types of cells but also to integrate topographic control over cell alignment with the design of scaffolds for tissue engineering purposes, such as scaffolds for reconnecting severed muscle tissue [54]. Taken together, the MST is a versatile and effective method to prepare the multi-structure and function of alginate fibers. However, the mechanical performance of the primary fiber was poor because of the lack of stretching, and it was just suitable for small batch production.

## 3. Physicochemical Properties of Calcium Alginate Fiber

### 3.1. Mechanical Performance

**Figure 9:**
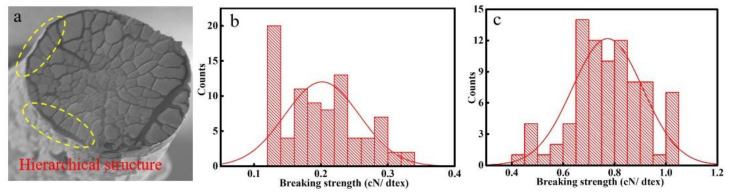
(**a**) SEM images of the hierarchical structure in the alginate fiber, Breaking strength of alginate fiber with the hierarchical structure (**b**) and without the hierarchical structure (**c**).

Calcium alginate fiber and its nonwoven fabric are ideal materials in the field of textiles due to the abundant marine resource and being environmentally friendly. Among them, the mechanical performance of fiber is the focal spot, which directly affects its range of application. The well-known setback of alginate fiber fabricated by different techniques is the formation rate is too fast to control, resulting in uneven structure in the fibers. The prevalence of structural defects of fiber can easily cause stress concentration and lead to fiber failure. A slower gelation and formation rate is an effective method to optimize the fiber structure and improve mechanical performance. Based on this, some researchers have resorted to a calcium salt composite solution with different solubility by choosing the compound coagulation bath system to retard the formation rate of fiber [55]. That was the original calcium chloride (CaCl_2_) solution combined with the indissolvable calcium salt solution to reduce free calcium cation concentration. Some other scholars chose other metal cations with better gelation ability to tune the ion exchange rate with alginate polymer, which retarded the Ca–Na cross-linking rate and achieved the homogeneous structure of metal-alginate fiber, following the order of the metal ion exchange rate: Pb^2+^ > Cu^2+^ > Cd^2+^ > Ba^2+^ > Sr^2+^ > Ca^2+^ > Co^2+^ ≈ Ni^2+^ ≈ Zn^2+^ > Mn^2+^ [56]. These works aimed to decrease calcium ion content for prolonging the formation rate of fiber, but the longer formation time resulted in forming a hierarchical structure in the fiber, as shown in Figure 9. The breaking strength of alginate fiber without the hierarchical structure is larger than that of the hierarchical structure. That was not beneficial for the improvement of the mechanical performance of the fiber.

**Figure 10:**
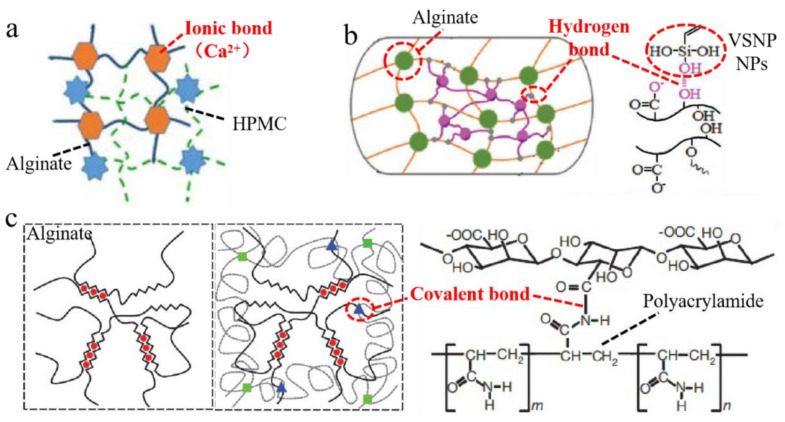
Introduction of (**a**) ionic bond, (**b**) hydrogen bond and (**c**) covalent bond in the alginate fiber.

While the above works succeed in retarding the formation rate of fiber by reducing the Ca–Na ion exchange rate, and decreasing the uneven structure in the fiber, the prolonged formation time caused another problem, delamination between the core and sheath layers of fiber. Some other researchers focused on the incorporation of reinforced materials in the spinning solution, which helped to strengthen the cross-linking interaction between the fiber molecular chain. The popular doping additive materials include the hydrophilic polymers such as collagen [45,57], chitosan [58,59], cellulose nanocrystal [40,60] and its derivative [55] or the nanoparticles including silica [61,62], hydroxyapatite [63,64] and graphene [42,65]. These strategies introduce extra hydrogen bond, ionic bond or covalent bond in the alginate fiber. For example, doping the hydroxypropyl methylcellulose (HPMC), the inorganic nanoparticles (VSNP NPs) or polyacrylamide, respectively, in the solution can improve the breaking strength and stretching performance of the fiber, as shown in Figure 10 [43,46,66]. Though these strategies yielded an improved result that the breaking strength of calcium alginate fiber was about 2.0 cN/dtex, a 30% increase, researchers are still not satisfied with the processing requirement, when compared with the common cotton or synthetic fiber.

Hence, to eliminate the defect in fiber, it is significant to investigate and disclose the formation mechanism of fiber. What is more, the impact of defects on fibrous mechanical performance is also needed to be further studied. Controlling the formation rate and improving the uniformity of fiber is the key to enhancing fibrous mechanical properties.

### 3.2. Moisture Absorption and Biological Compatibility Performance

Alginate is one of the most preferred biomaterials as an abundant natural biopolymer, which was approved by the United States Food and Drugs Administration [3,67]. Calcium alginate fiber is nontoxic and has high hygroscopicity and biocompatibility, making it a perfect candidate for biomaterials such as wound dressing and tissue engineering scaffolds [68,69]. Significantly, alginate fiber can imitate the physicochemical environment of tissue and its degradable product in vivo can be efficiently cleared by the renal, which is beneficial for tissue repair and regeneration. As an ideal biomaterial, the hygroscopic properties and biocompatibility of alginate fiber have become the research focus.

**Figure 11:**
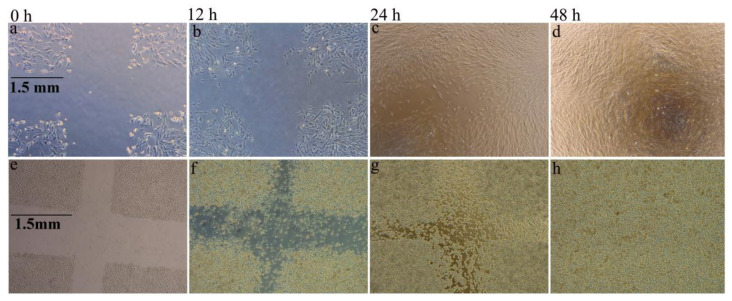
The migration of fibroblasts (**a**–**d**) and keratinocytes (**e**–**h**) covered on the simulated scratch wound [15,58].

The alginate fiber has excellent moisture absorption capacity, which can absorb 10 times more than its own weight of water [70,71]. Its molecular chain displays abundant hydrophilic groups including -OH and -COOH groups and can combine a great deal of polarized water molecules. This high absorbency can keep the wound moisture and reduce local pain by providing a cooling effect. It does not adhere to the wound bed and the new granulation tissue will not be affected by washing away the alginate fiber, which can form a self-adherence process in the peri-wound area with a good cover of the infected area. Therefore, the remarkable hygroscopicity of alginate fiber is consistent with the modern theory of moist wound healing [28], holding the potential as a candidate for wound dressing. Moreover, biocompatibility is the other critical factor for its utilization as a biomaterial. It was demonstrated that this fiber can degrade into small molecules including the polysaccharide or its derivative and then be naturally excreted from the body through metabolism [52], during which no hazardous substances remain in the human. Some researchers [15,62] used alginate fibrous dressing to cure the simulated scratch wound, and the result is shown in Figure 11. The simulated scratch wound formed by culturing the fibroblasts and keratinocytes in a culture plate and then created with 20 µL micropipette tips, was completely covered and healed after 48 h, respectively. This result proved that the dressing facilitates the proliferation and migration of fibroblasts and keratinocytes, implying no significant cytotoxicity of the alginate fiber dressing [62]. Furthermore, other works have also implanted the alginate fibrous scaffold into a rat model of *S. aureus* bacterial infected wound, and the histological analysis indicated that the alginate fibrous scaffold acted a positive effect on the acceleration of wound healing [52]. The healing process involved the overlapping phases of inflammation, cell migration and proliferation, neo-vascularization and extracellular matrix production, all of which certified the biocompatibility of alginate fiber. Taken together, the preferable hygroscopic and biocompatible properties of alginate fiber make it become a hot biomaterial.

### 3.3. Flame Retardant Property

**Figure 12:**
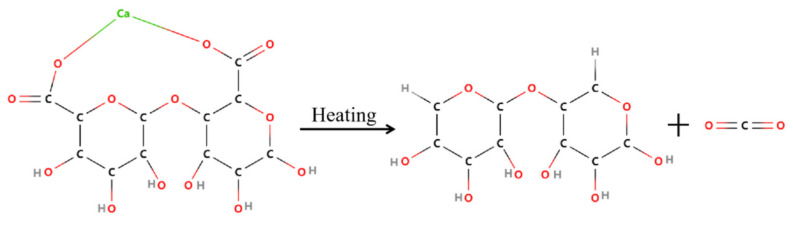
Schematic diagram of thermal decomposition of alginate fiber.

Alginate can cross-link with most of the divalent or trivalent metal ions such as Ca^2+^ by the supramolecular interaction sites to achieve outstanding inherent flame retardancy, because of the inert metal ions during the heating. Calcium alginate fiber as a common alginate-based material exhibited excellent intrinsic flame retardant properties due to the presence of calcium cation and abundant oxygen atoms in its molecule, as displayed in Figure 12. The fiber forms calcium salt and releases the noncombustible carbon dioxide (CO_2_) gas and gaseous water vapor in burning, which cover the fiber surface and then impede the penetration of oxygen (O_2_) and the heat, which will effectively retard the further combustion. Furthermore, the heat release rate, the total heat release, the smoke production rate and the total smoke production of the calcium alginate fiber are far less than other materials in the whole burning [72], making it an ideal candidate for the fireproof textile and furniture construction material.

**Figure 13:**
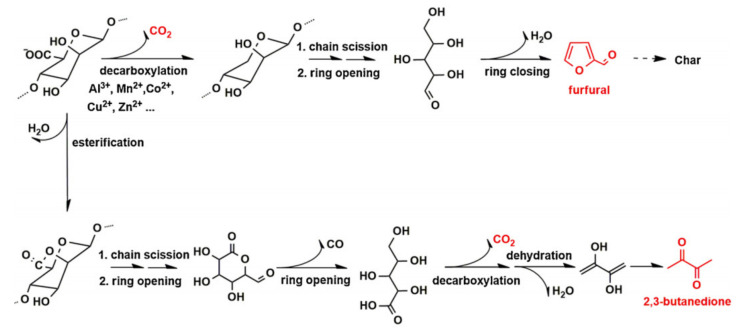
The proposed pyrolysis pathways of alginate fiber.

Xia et al. [73] and Zhu et al. [74] have demonstrated the wet spun alginate fiber to have outstanding flame retardancy with a limiting oxygen index (LOI) as high as 48%, while that of cotton and viscose fiber is about 18% and 20%, respectively. Moreover, the time to ignition (TTI) of alginate is 142 s, which is much longer than any other fibers. Thus, alginate as a kind of functional and high-value material was used to construct fire-resistant textile or as a component and modifier of fireproof coating to reduce the flammability of textile. Driven by this encouraging result, a number of works have investigated the pyrolysis behavior of alginate fiber by the measurements of synchronous thermal analysis, then studied its flame retardant properties by carrying out the vertical flame test (VFT), UL 94 and cone calorimetry test (CCT), and finally disclosed and established the fireproof mechanism by the combustion product analysis of Thermogravimetric Infrared Spectroscopy (TG-IR) and Pyrolysis Gas Chromatography–Mass Spectrometry (PY-GC–MS). As exhibited in Figure 13, all the results indicate that the alginate fiber pyrolyzes according to the decarboxylation or esterification pathway in the burning, in which 2,3-butanedione and furfural are the main combustion compounds [3,73,74]. There were fewer volatile combustible matter, heat and smoke in burning, illustrating the excellent flame retardancy and environmental friendless of alginate fiber compared with other nonflammable fiber.

## 4. The Application of Calcium Alginate Fiber

### 4.1. Wound Dressing

The treatment of acute and chronic wounds with inflammation caused by burns, scalds or the complications of chronic diseases, is an urgent medical need [75,76,77]. Traditional wound dressing such as cotton gauze, petrolatum gauze, bandages and so forth, has displayed a serious barrier to wound regeneration. The fibrous dressing absorbs the wound exudates and then helps them to evaporate to keep the wound drying, which can prevent the contamination with bacteria and pathogens [78,79]. The dressing protects the wound from external disturbance, but the dry wound is not helpful in the proliferation and migration of skin cells. In addition, the new granulation tissue of the dry wound can be torn easily in the dressing replaces, which may trigger secondary damage. The dry dressing cannot provide and maintain the needed temperature of the wound, making it retard the wound healing. In contrast, the novel modern dressings such as alginate dressing (e.g., hydrogel dressing, the porous scaffold, the fibrous or fabric dressing) can provide and keep a moist wound environment, and then accelerate the migration and re-epithelialization of epithelial cells of the wound, which was a benefit for the wound healing.

Alginate wound dressing can appear in the form of a hydrogel, fiber, nonwoven fabric, freeze-dried scaffold and foam, all of which are prepared by Ca–Na ionic cross-linking interaction. Calcium alginate fiber or the corresponding nonwoven fabric employed as a kind of typical wound dressing, can absorb the wound exudates and then formed gel to keep the wound wet. That provided a safe, sealed and physiological microenvironment, and minimized the bacterial infection possibility around the wound. Furthermore, the hypoxia microenvironment stimulates angiogenesis and facilitates cells to secret the growth factor, which promotes the new granulation tissue formation and accelerated wound healing [80]. However, the only gelation function of the original alginate fiber dressing cannot satisfy the actual clinical needs. A variety of multi-functional and bioactive wound dressings are an urgent clinical need.

For enriching the function of alginate fiber dressing, many works have combined alginate with some special therapeutic effects of drugs [81], various growth factors [82], metal ions [83] and polymers [84,85]. For instance, Tang et al. [86] prepared the functional alginate dressing by incorporating quaternized chitosan (hydroxypropyltrimethyl ammonium chloride chitosan) and magnesium (Mg) in the spinning solution to cure diabetic foot ulcers. The modified chitosan and Mg metal particles can effectively eradicate methicillin-resistant *Staphylococcus aureus* and methicillin-resistant *Staphylococcus epidermidis*, displaying an outstanding antibacterial ability. The results of in vivo microbiological and histological analysis illustrated that alginate dressing containing the functional addition facilitated the migration of human dermal fibroblasts and human umbilical vein endothelial cells, which stimulated angiogenesis and accelerated wound healing. In addition, Vieira et al. [84] immobilized the papain on alginate fiber wound dressing to endow the dressing with excellent wound healing ability, which was also capable of promoting the debridement of devitalized or necrotic tissues. The added papain can stimulate the production of cytokines to promote the local cell multiplication and narrow the wound edge, which was a benefit for reducing the scar and assisting wound healing. In conclusion, the incorporation of multi-functional additives in alginate fiber wound dressing has yielded good progress and become the future development trend.

### 4.2. Tissue Engineering Scaffold

The other major application of calcium alginate-based fiber is tissue engineering scaffold, as a cost-effective material for cell immobilization and encapsulation. Compared to the wound dressing, the tissue engineering scaffold has additional requirements such as serving as an extracellular medium matrix to support cell growth, migration, differentiation, and eventually cell normal function. Multiple pieces of literature reported alginate hydrogel serving as the tissue engineering scaffold, in a bulky form [87], or as an ink to be printed into a designed form [88], while investigation on calcium alginate fiber is also underway extensively. The fiber provides a few features that hydrogel does not have. As the assembly of fibers, the space between fibers allows a fast transport of nutrients and oxygen to the regeneration site and quick release of waste [89]. Additionally, the manipulation of fiber orientation into an aligned form mimics the physiological environment, especially for the purpose to cue multiple cells to arrange into an aligned form, including muscle cell [90] or neuron [91], where the pattern is crucial for the activation of normal physiological function.

There are a few challenges that remained. Though the microfluidic spinning technique described above was used by several reports as the bioactive scaffold, the general problem of micrometer-diameter fiber is that the space between them is too large for cells, which may require extra time for the cell to fill the space. People turn to electrospinning to reduce the diameter of the fiber to match the cell size. However, there are more challenges, particularly for alginate in electrospinning. Alginate is an ionic polymer that hydrolyzes in water to increase its conductivity, making the solution extremely difficult to electrospin because of the risk of a power surge. Moreover, its lack of shear strength in the sodium salt form and being too dense in the calcium salt form makes it difficult to electrospin. Additionally, there is a very limited choice of solvents because of the hydrophilic nature of the alginate molecule, leaving the only choice to be water with a high boiling point. Many researchers overcome this problem by adding other water-soluble polymers to help overcome mentioned problems. Commonly used polymers include PCL [92], chitosan [93], collagen, gelatin, and PEO [94] to either make electrospinning feasible or bring additional properties needed in tissue engineering. Researchers have used these fibers for the regeneration of bone, muscle, neuron, and skin.

As a tissue engineering fiber, the alginate was used to carry different drugs to facilitate the regeneration speed, including small chemical drugs, nucleic acid [95] and protein [96]. Additionally, the cations in the calcium alginate fiber can be substituted with other bivalent charge cations that have physiological functions, such as Zn^2+^, Mg^2+^ and Cu^2+^. Those bivalent charge cations can replace calcium without destroying its gelation property. For example, calcium can be replaced with copper cations [97]. A small amount of copper ions was proven to have a profound effect in enhancing tissue sealing and repair, because of the using angiogenesis ability of copper to help form new blood vessels, and its photo-thermal ability to help the fiber to heat up. Additionally, the calcium in the fiber can be the nucleation site for mineralization, which is the key step to introducing hydroxyapatite to the scaffold and using it to enhance the osteogenesis ability [94,98].

### 4.3. Smart Material

**Figure 14:**
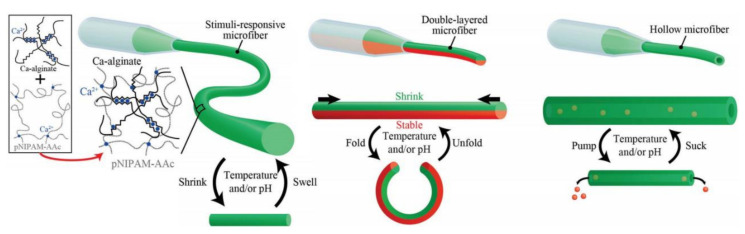
Schematic diagram of a pH/temperature-responsive alginate microfiber with single or double layered structure fabricated by the microfluidic system.

With the development of smart materials, some researchers used the active groups of alginate molecular chains such as -OH and -COOH groups to cross-link with polymers to obtain special intelligent response performance. The covalent compounds such as polyacrylamide or poly (vinyl alcohol) were incorporated into the alginate molecular chain to introduce the covalent bond for fabricating the highly stretchable fiber strain sensor utilization as the ionic skin, wearable and implantable sensors for innovative electronics [99,100]. The concomitant ionic bond and covalent bond endowed the alginate fiber with remarkable mechanical performance especially its high stretchability based on the energy dissipation and deformation hysteresis [101,102]. The fiber is full of metal cations, thus the fiber can have a desirable electrical conductivity, making it a potential platform for smart wearable and implantable innovative electronics. In addition, other researchers used alginate fiber to fabricate the soft actuators and robots [103], thermosensitivity [104] and pH-sensitivity sensor [105] by the addition of some functional temperature/pH sensitive materials as exhibited in Figure 14, which were demonstrated to achieve a preferable intelligent response effect to the surrounding environment. Taken together, it is believed that the alginate fiber displays the fabrication possibilities for novel components of soft actuators and micropumps, as well as smart wearable devices for various sensors.

### 4.4. Fire-Resistant Material

With a tightening low-carbon policy, bio-based materials have received more attention than petroleum-based materials. The commercial fire-resistant materials receive flameproof treatment by using halogen or phosphorus-based flame retardants. However, the fabric treated by the fire-retardant finishing will release some toxic volatile gaseous compounds in the thermal degradation process, which is harmful to human health and violates the eco-friendly concept. What is more, the flame retardancy of that fabric is not durable, decreasing with the increase in usage times. Therefore, the bio-based flame-resistant material has become a research hotspot due to its sustainability, environmental friendliness and recyclability, such as protein, chitosan, DNA, starch, phytic acid and so forth. Among them, the abundant marine material has been widely used to prepare film [106,107], hydrogel [108], fiber [109,110,111], fabric [74,112] and aerogel [113,114,115] for fire prevention. Alginate fiber as an intrinsic fireproof material has become an ideal flame-resistant textile material application in the field of industrial and academic.

**Figure 15:**
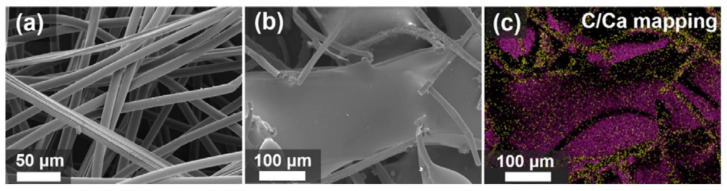
SEM images of polyester/alginate mixed fiber (**a**) before and (**b**) after the flammability test, and (**c**) EDX element mapping image (purple for carbon mainly from melt polyesters and yellow for calcium in fibrous charred alginate).

The environmentally sustainable flame retardants for textiles and inherently flame-retardant fiber materials thereby have become mainstream. There were some reports that illustrate that the toxic gaseous compound including CO, CO_2_ and smoke production rates of the alginate fiber in burning were very little, which was in accordance with a low carbon concept [116,117]. Recently, the alginate fibers were mixed with viscose fibers to produce the nonflammable composite nonwoven felt by the acupuncture technology, serving as the doll filling materials [107]. The alginate fibers and cotton fibers were twisted into yarns at various mass ratios to fabricate the eco-friendly flame-retardant textile fabric by the knitting or weaving technique, which can be applied in the fire-fighter uniform. Those works have achieved the great flame retarding effect and the introduction of alginate fiber was demonstrated to improve the flame retardancy of fabric. Although the alginate fiber displayed an excellent flame retardant performance, it experienced a minor afterglow phenomenon after removal from the fire, which was a drawback for its fireproof abilities. To address this hurdle, some researchers combined the alginate fiber with some synthetic fibers such as polyester to obtain high flame resistance materials [118]. The mixed polyester fiber can form the molten drop in burning and then reduce the afterglow of alginate fiber. As shown in Figure 15, SEM images of the alginate composite fibrous mat before and after the burning, illustrated the droplet covered on the fiber to inhibit the afterglow. Facile blending has become an effective approach to obtaining a self-extinguishing composite alginate fibrous mat or fabric. However, the smoldering process of the original alginate fiber has not been eliminated, and the incorporation of flammable petroleum-based fiber decreased the fire-resistant property and produced some toxic gaseous products in burning, which limited its application as a flame retardant protective material.

Hence, it is urgent to eliminate the smoldering combustion behavior of alginate fiber and the simple mixture of other synthetic fibers is not a good strategy. The molecular chain of alginate fiber displays a good deal of active groups, which can be easily modified and grafted with the corresponding groups to optimize the flame retardant property of alginate fiber in the future.

## 5. Conclusions and Future

Compared to the numerous research and review articles on alginate hydrogel, a review on fabrication, physicochemical performance and application properties of alginate fiber is very rare, resulting in the lack of corresponding knowledge. Nevertheless, alginate fiber, as a family of emerging advanced functional materials, has demonstrated great utility and potential, particularly in the wound healing, tissue engineering and flame retardancy fields. The most attractive features of alginate fiber for these applications are attributed to its excellent moisture absorption performance, biocompatibility and intrinsic flame retardant property. Therefore, this work mainly summarized the fabrication of alginate fiber and compared their respective characteristics, and then analyzed the corresponding application properties.

As a wound dressing or tissue engineering scaffold, alginate fiber has a track record of clinical safety and was safely implanted in various hosts including the islet transplantation for treatment of type 1 diabetes, chondrocyte transplantation for treatment of urinary incontinence and vesicoureteral reflux, and the infected wound transplantation for healing of the inflammatory. Although, alginate fiber is widely used in the field of biomaterials and is likely to evolve considerably. It already plays a fairly passive role during treatment due to a shortage of multi-functions. Future dressing or tissue scaffold will likely play a much more active role, which requires the material itself to cure and accelerate the wound healing or repairing impaired tissue in the host. One or more bioactive agents such as proteins, DNA, antibiotics and other polymers that reduce inflammation and promote bone growth, can be incorporated into the alginate fiber as a facile and efficient method to endow it with a multi-functional design, which has been proven to be the future trend.

In addition, the alginate fiber as a burgeoning bio-based flame retardant material has proven to be the future trend, due to the rare limited significant toxicity combustion product and the intrinsic eco-friendly behavior. However, the decrease in its minor afterglow profile and the enhancement of mechanical properties is an urgent need before its widespread application in the fireproof construction and building field. As one looks to the future, the elimination of fibrous structure irregularity is the future development. For addressing the hurdle of the afterglow behavior of alginate fiber in burning, the grafted modification of the alginate molecular chain is probably a new and efficient strategy. In conclusion, alginate fiber as a novel eco-friendly bio-based material was proven to have a broad application prospect in the biomaterial and fireproof construction fields.

## Data Availability

The study did not report any data.
